# Success and Failure of Parliamentary Motions: A Social Dilemma Approach

**DOI:** 10.1371/journal.pone.0133510

**Published:** 2015-08-28

**Authors:** Roel Popping, Rafael Wittek

**Affiliations:** University of Groningen, Department of Sociology, Groningen, The Netherlands; Southwest University, CHINA

## Abstract

Parliamentary motions are a vital and frequently used element of political control in democratic regimes. Despite their high incidence and potential impact on the political fate of a government and its policies, we know relatively little about the conditions under which parliamentary motions are likely to be accepted or rejected. Current collective decision-making models use a *voting power* framework in which power and influence of the involved parties are the main predictors. We propose an alternative, *social dilemma* approach, according to which a motion’s likelihood to be accepted depends on the severity of the social dilemma underlying the decision issue. Actor- and dilemma-centered hypotheses are developed and tested with data from a stratified random sample of 822 motions that have been voted upon in the Dutch Parliament between September 2009 and February 2011. The social dilemma structure of each motion is extracted through content coding, applying a cognitive mapping technique developed by Anthony, Heckathorn and Maser. Logistic regression analyses are in line with both, actor-centered and social-dilemma centered approaches, though the latter show stronger effect sizes. Motions have a lower chance to be accepted if voting potential is low, the proposer is not from the voting party, and if the problem underlying the motion reflects a prisoner’s dilemma or a pure competition game as compared to a coordination game. The number of proposing parties or a battle of the sexes structure does not significantly affect the outcome.

## Introduction

Parliamentary motions are a vital and frequently used element of political control in democratic regimes. For example, in the Dutch parliament, an average of 830 motions have been proposed on a yearly basis in the period from 1998 to 2006 [1]. Parliamentary motions are formal requests to the government to take a specific action [2]. They can be proposed by any Member of Parliament, can cover a wide range of issues (e.g. legislative, budgetary or petitionary), and they can have severe consequences. For example, in extreme cases the passing or rejection of a motion can be decisive for the continuation or termination of governments: of a total of 1099 no confidence motions that were proposed in 20 advanced parliamentary democracies in the period from 1960 to 2008, 55 (5%) resulted in the termination of government [3].

Despite their high incidence and potential impact on the political fate of a government and its policies, we know relatively little about the conditions under which parliamentary motions are likely to be accepted or rejected. The present study wants to answer this question.

Explaining and predicting the outcomes of political negotiations is of course a core objective of research on collective decision-making since more than half a century. In most of the models developed in this tradition, salience, power and influence of the involved parties play a crucial role [4, 5]. We argue that much is to be gained by complementing this *voting power* framework with a *social dilemma* approach [6, 7]. A social dilemma approach uses cognitive mapping techniques to uncover the type of the social dilemmas behind a decision issue. Within this alternative framework, a motion’s likelihood to be accepted depends on the severity of the social dilemma underlying the decision issue.

Our study makes three distinct contributions to current scholarship. First, it is the first to elaborate the theoretical foundations of a social dilemma explanation of the success of parliamentary motions. Second, we demonstrate the feasibility of applying cognitive mapping techniques to extract different types of social dilemmas behind parliamentary motions. Third, our study is the first to empirically test both voting power and social dilemma explanations in a sizeable sample of parliamentary motions in the Dutch Parliament.

The next section provides some background information on parliamentary motions in the Netherlands. After that, the theoretical framework is introduced and empirically testable hypotheses are derived. The following section describes the data and the cognitive mapping techniques that were used to extract social dilemmas from the documents containing the motions. The results of our empirical tests are presented after that. The final section concludes.

## Parliamentary Motions in Dutch Politics

The Netherlands has a bicameral parliament, consisting of the Senate and the House of Representatives. The latter is the main chamber. It has 150 seats, which are distributed according to a party-list proportional representation. The House of Representatives has the right to propose and discuss legislation (which upon approval by its majority is sent to the Senate). It also has the right of interpellation, and the right to propose amendments and motions.

**Fig 1:**
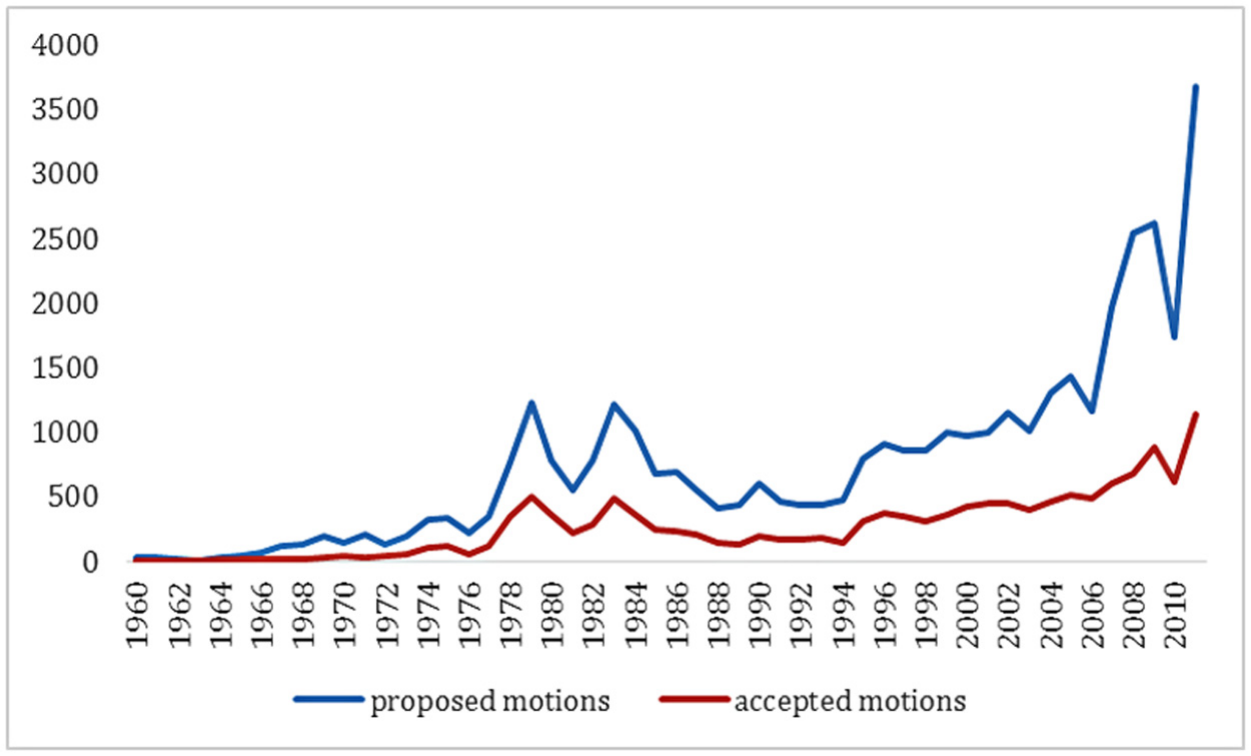
Number of Proposed and Accepted Motions in Dutch Parliament (1960–2011). Source: http://www.politiekcompendium.nl/9351000/1f/j9vvh40co5zodus/vh4vaj9dvdkx and letter 2011–2000570709 Minister of Governance and Kingdom Relations, December 15 2011.

Motions are an important and frequently used instrument in Dutch Parliament. In 2006, 84% of the members of the Dutch House of Representatives indicated that they consider the motion as an essential instrument in their work [8]. The use of motions increased through time. Whereas the absolute number of proposed motions was 24 in 1960, this figure reached a record total of 3679 in 2011 (see Fig 1).

A *parliamentary motion* is an official statement in which (one or more) members of a parliament—usually as representative[s] of one or more parties—ask the government to carry out some specific action, usually a change in policy. Motions can also be directed towards the House itself, but this class of motions is beyond the scope of this study. Since the introduction of dualism (i.e. the separation of powers between parliament and cabinet) into Dutch politics, one of the functions of parliament is to control the government. As a consequence, the initiative for motions does not only come from the opposition, but also from parties of the governing coalition.

Two specific types of motions need to be distinguished. *Motions of disapproval* imply the rejection of some specific government policy, but their outcome is not binding—the government is not obliged to take action, even if a majority accepted the motion. *Motions of distrust* express fundamental doubts about a policy. If approved by a majority, they are binding: the Secretary responsible for the specific policy or the government as a whole is dismissed.

The procedure of proposing a motion consists of six main steps. In a *first step*, one or more members of the House of Representatives can propose a motion, if at least five members of the House support the submission. A motion usually contains four elements: an introduction, a consideration, a request or judgment and a formal closure (see Appendix for examples). The *second step* consists in the discussion in the House, when its members debate about the motion. During this step, representatives of each party explain the position taken by their party. In a *third step*, the respective Secretary comments on what has been brought forward. In a *fourth step*, the representatives get the opportunity to comment, based on this new information. If the proposer of the motion is satisfied with the answer, the motion might be withdrawn. This happened regularly in cases where the Secretary agreed to carry out the requested actions. The *fifth step* takes place after the discussion finished and before the voting: the Secretary makes a statement concerning the request in the motion. The statement consists in either declaring the motion as unacceptable, advising against it, or leaving the evaluation to the House. In the Netherlands it is obliged to make this statement. In the *final step*, in case the motion is not withdrawn, the House votes in favor or against the motion. If the majority of the members (half of the votes plus one) support the motion, this results in a Statement by the House. This implies that each party has to carry out two types of negotiations: it has to negotiate with other parties in order to get sufficient votes, and it has to negotiate with the government in order to get her demands realized.

## Theoretical background

Research on parliamentary motions is scarce and the few recent studies addressing them focus on the fact that they are not always used for the purpose for which they are designed, i.e. controlling task of the House of Representatives. For example, in [3] and [9] it emphasized that parties often use motions as a symbolic instrument to signal their policy position other parties and potential voters. Put differently: the main motive behind proposing this kind of motion is not to get it passed, but to make a statement. This type of symbolic motion has also been denoted as “exclamation mark motions”, as opposed to “question mark notions” [9]. In such cases, parties propose a motion to make visible that they do not agree with a specific policy, and they know that the motion will not pass the vote.

Our study complements the growing research interest in motions by shifting the emphasis from the signaling character of motions to their success or failure in the voting stage, A variety of models have been proposed to explain the outcomes of collective decision-making processes [10], and the field has made considerable progress since the coalition models as they were introduced in the early 1970s [11]. The current toolkit meanwhile comprises a broad range of cooperative and non-cooperative models for both the bargaining and the voting stage [5]. They have been applied to a broad range of political or economic negotiation settings, like collective labor agreements, city council, coalition negotiations or European Union decision-making. With the proportion of correctly predicted outcomes often reaching 80% and more, these models can also be considered to be quite successful.

Existing models so far pay much attention to three critical elements of the negotiation: characteristics of (and relations between) the involved parties (like their policy position, power, coalitions, and the salience of the issue); the decision issue (like the range of potential decision outcomes); and the institutional context (like procedural rules specifying how to aggregate votes). The existing models do not take into account the types of conflict. We first present some straightforward hypotheses from this voting power perspective, before introducing our social dilemma approach.

### Voting Power Perspective

Up until now, investigations on motions focus on the consequences of these motions, rather than on modeling their success or failure. For example, Williams [4] uses signaling theory to explain increases and decreases of vote shares for coalition and opposition after no-confidence motions in 20 parliamentary democracies. He argues that no-confidence motions enable opposition parties to signal their power and enlarge their vote shares, even if such motions may have a low probability of success. In [12] this analysis is extended to show that as a consequence of no confidence motions, the position of political parties shifts away from the government’s position, in particular if confronted with negative signals about government performance. Focusing on the Dutch case and drawing on theories of political space, In [1] motions of the period from 1998–2006 are used to show that the entry of a new right wing party resulted in an increase of the number of significant lines of conflict.

More generally, when applied to modeling success or failure of motions, the voting power perspective suggests a linear relationship between the relative power of the proposing camp and the likelihood that a majority of the members of the House will vote in favor of the motion. Three different indicators can be used to assess the relative power of a party.

First, the coalition has a structural power advantage over the opposition, since the government has to advice on the motion. The government can follow the proposing party or disagree with the motion. The likelihood that the government agrees with the motion is higher if a member of a coalition party proposes the motion.

Second, power of an initiating party increases to the degree that it succeeds in mobilizing support for the motion among other parties already during the proposal stage. The number of parties endorsing it is an important indicator for the amount of support a motion can build. Though some parties might only represent a small proportion of the electorate, the absolute number of parties endorsing it reflects to what degree it may draw on support from heterogeneous groups in society. Hence, the higher the number of parties that propose the motion, the higher the likelihood that the motion will be accepted.

Third, in the Dutch parliament, as in many other countries, parties usually vote as a block, i.e. when a party supports a motion, all members of that party in parliament will vote in its favor [13]. In political science this is used in studies on voting behavior of individual parties. The interaction within the parliament can be analyzed in terms of negotiations between parties, rather than among individual members of parliament [14, 15]. In fact, Dutch governing parties tend to protect their party’s interests rather than the interests of the government as a whole [15]. If a governing party in the coalition is not satisfied with a government agreement, the party tries to find partners in the opposition who support their position in order to change government policy. Hence, the number of proposing parties allows inferring the so-called voting potential of a motion, i.e., the number of favorable votes a motion might elicit. We are not aware of studies in which this view is surveyed.

In sum, a voting power perspective suggests the following general hypothesis:

*Hypothesis 1 (Voting Power)*: The likelihood that a motion is accepted increases (a) if it is proposed by a member of a coalition party, (b) the higher the number of parties supporting it, or (c) the higher the voting potential.

### Social Dilemma Perspective

**Fig 2:**
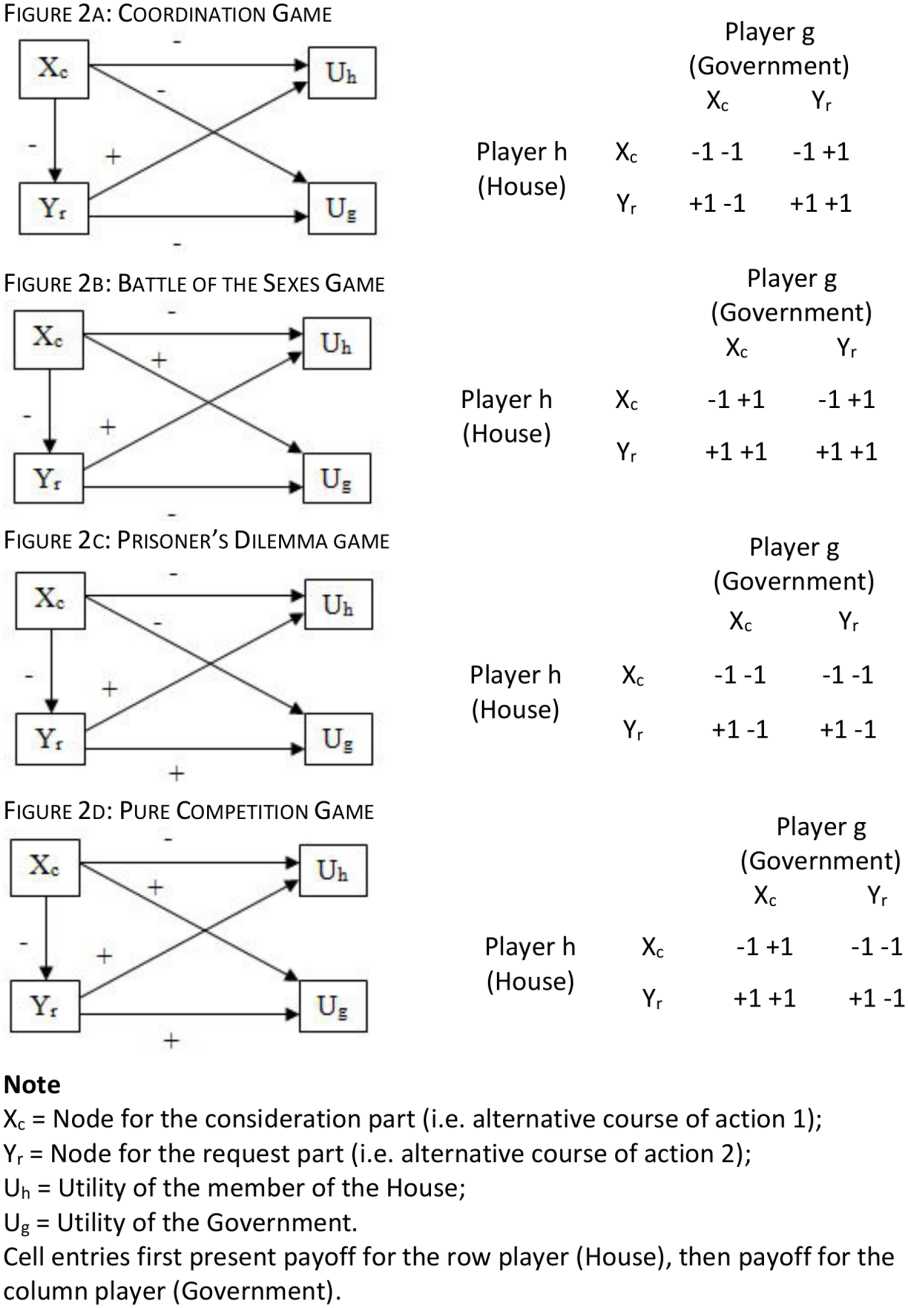
Graphical Representation of Different Types of Social Dilemmas and payoff matrices. Note: X_c_ = Node for the consideration part (i.e. alternative course of action 1); Y_r_ = Node for the request part (i.e. alternative course of action 2); U_h_ = Utility of the member of the House; U_g_ = Utility of the Government. Cell entries first present payoff for the row player (House), then payoff for the column player (Government).

Whereas the voting power perspective focuses on characteristics of the involved players and their relations, a social dilemma perspective shifts the attention to the content of the motion. Unlike earlier research on the content of motions [1], the focus is not on “topics”, but on the structural properties of the problem described in the motion. Building on earlier work [6, 7], we suggest that problems differ in terms of their severity. More specifically, we argue that problem severity can be conceptualized in terms of different types of social dilemmas underlying a parliamentary motion. A social dilemma refers to a situation in which all involved players would be better off if everyone chose the cooperative option, but where it is rational for each individual player to defect. There is a wide range of different types of social dilemmas [16]. Depending on the payoff structure, some of these dilemmas are “easier” to solve than others. For this study, we distinguish between four types of games, which can be ordered on a continuum of increasing severity. Fig 2 contains the graphical representations of the dilemmas that will be discussed hereafter and the payoff matrices for each of them.

The first and least severe type is a *coordination game*. Strictly speaking, coordination games are not social dilemmas: due to the payoff structure of coordination games, the involved actors do not have competing interests. Both parties strive for the same goal and need each other to attain it. In its simplest form, a coordination problem is given if two actors can choose between two alternative courses of action X or Y. If the actors both choose X or if they both choose Y, both receive the same high payoff. If the one actor chooses X and the other Y, they both receive a low payoff. Given this payoff structure, both actors are indifferent with regard to the two options X or Y, as long as they can be sure that the other actor opts for the analogous alternative. The problem is that they do not know which alternative the other actor will choose. If they have this information, the solution to this coordination game is self-enforcing, because it is in each actor’s best interest to choose the same option that the other party chooses. A key feature of coordination problems is that there are no conflicting interests separating both parties. Both parties are willing to cooperate, if they only knew in what way [17]. Because the interests of both parties point into the same direction, this kind of problem is relatively easy to solve.

The second type of problem can be characterized as a *battle of the sexes game* [18]. It is similar to a coordination game in that both players have an interest in choosing the same alternative, i.e. they are better off if they coordinate. But unlike in a coordination game, battle of the sexes games contain some degree of mixed-motive interdependence: the payoffs in the two coordination solutions are asymmetric. Player 1 benefits slightly more if solution X is chosen, whereas Player 2 benefits slightly more if solution Y is chosen. Hence, it is in the best interest of both to coordinate on one of the two cooperative solutions, even if this means that one of the players will receive a higher payoff than the other one. Consequently, battle of the sexes problems can be located at the low end of the problem severity continuum, since it strongly resembles a pure coordination game.

The third class of problems resulting from mixed-motive interdependence is the well-known *prisoner’s dilemma*. Here, coordinating for a cooperative solution implies that players would forgo a potential gain that might be realized if one would defect but the other player would cooperate [18, 19, 20].

The fourth type of problem is a situation of *pure competition* [21]. Here, the interests of both parties are completely opposed, i.e., one party can only obtain positive utilities at the expense of the other party. Since pure competition problems are zero-sum games, there is no incentive to avoid or settle a conflict. Consequently, pure competition problems are the most severe ones in our typology.

*Hypothesis 2 (Social Dilemmas)*: The likelihood that a motion is accepted decreases with the severity of the underlying social dilemma, being highest for motions of the coordination and battle of the sexes type, and lowest for motions of the prisoner’s dilemma and pure competition type.

## Data and Method

Given their highly structured and codified form and the presence of a clear (binary) outcome in the form of a vote, motions are particularly well suited to study the link between variations in the type of social dilemma and of collective decision making. Unlike transcripts of unstructured debates or minutes, the content of a motion follows a predefined structure; the set of relevant players is unambiguous, as is their position in the negotiations. Taken together, this considerably reduces the complexities and complications resulting from extracting social dilemmas from more unstructured texts [7].

### Sample

The present data set contains all motions that have been proposed in the Dutch Parliament between September 2009 and February 20, 2010 (the day the government resigned) or between September 2010 and February 2011. These time intervals are the ones in which the House deals with the budget for each department. If the House wants to influence the government’s policy, then the period from September to February is best suited for this purpose.

Three types of motions were excluded from the analysis. First, motions proposed by an outgoing government. Such motions may systematically differ from other motions, because of the many additional restrictions governments in such a situation have to face. Second, motions directed at the House itself, since our focus on social dilemmas requires the government as a player and target of a request. Third, motions withdrawn or expired before having been put to vote (n = 122).

During the two intervals subject to our study, 2602 motions have been proposed. Of these, 528 motions have been replaced, have expired or were withdrawn, leaving 2074 motions that have been put to vote. Mean acceptance rate during the whole period was 43.1%, varying from 42.5% during the first phase (2009–2010) and 43.7% during the second phase (2010–2011). Of these 2074 motions we coded a stratified random sample of 822 motions. Stratification is based on dividing the motions in 10 equally sized groups based on time of proposal. This should guarantee that motions from all debates are included in the sample and that also the number of motions per debate is taken into account.

### Extracting Social Dilemma Structures from Texts

Motions in the Dutch Parliament are highly structured texts that codify parts of a political negotiation. Their mechanistic and transparent nature considerably facilitates quantitative analysis [22]. They consist of an introduction, a consideration, a request or judgment and a formal closure. Consider the following example [23] (see also the examples in the Appendix):

“The House, having heard de deliberations, considering that in the master plan prison system 2009–2014 has also been recorded that some institutions having limited guarding (ILG) and some having very limited guarding (IVLG) will be closed, considering that from several (international) researches it appears that the chances on repetition by prisoners after dismissal from open and half-open, less protected institutions are lower than after dismissal from more closed and more protected institutions, moreover considering that places in ILG and IVLG are cheaper than places in closed institutions with a regular guard level, requests the government to postpone the planned closing of ILG and IVLG, and to investigate how these valuable places for the prison system can be remained preserved, and returns to the order of the day (Motion 24587, nr. 361).”

Building on work by Anthony, Heckathorn and Maser [6], we use cognitive mapping techniques to identify different types of social dilemmas in texts. A detailed description of how this approach can be applied to the coding of parliamentary motions is presented in [23]. Here, we sketch the general logic behind the approach. Several steps need to be conducted.

First, so-called *concept variables* need to be identified in the text. A concept variable is an entity that can take different values (e.g. ‘education’ can be high or low, a campaign can be successful or unsuccessful, etc.). Two types of concept variables are distinguished: a consideration part, and a request part: “The consideration part in a motion contains one or more descriptions of states of affairs and processes necessary to build up an argument, and judgments about both in order to express an opinion. The request part contains a judgment concerning the improved issue.” [23]. Note that one motion may contain more than one consideration part and request part, and therefore may contain more than one underlying social dilemma. In the example, the consideration concept variable is the closing of the prisons with (very) limited guarding—an act that the government intends to perform, according to the proposer. The request concept variable refers to postponing the decision and to maintain these prison places. More consideration parts are necessary to build up the argument. A small number of motions in our sample (n = 67) contains more than one request part. This happens if the proposer wants to indicate the steps in the policy process that are to be followed in case the motion will be executed. In her comment on the motion, the government always treats it as one entity and does not discuss separate parts. We therefore treat the consideration parts as a single concept variable. The same holds for the request parts.

Second, between these concepts we identify *causal relations* as they are explicated in the text. Since the consideration contains the reasoning leading to a request, the causal relation goes from the consideration part to the request part.

Third, for each causal statement, we identify which actors or *utility nodes* are affected by it, i.e. to what degree a concept variable is likely to result in a decrease or increase of their utility [6]. Hence, each of the two types of concept variables is related to both utility nodes, and the value of each relation can differ. Since an action is asked from the government in all of the motions in our sample, the government always represents one of the two utility nodes in a map. Here, a complication is that the motion may refer to “the government” as unitary actor, or (implicitly) address specific parts of the government, in which case the latter are taken as utility nodes. The other utility node represents the members of the House. Though this player could technically be seen as a unitary group, it is theoretically more adequate to disentangle this group in terms of the different political parties. This allows more fine-grained analyses, e.g. concerning the question to what degree the cognitive maps between the government and coalition parties differ from cognitive maps between the government and opposition parties. In the example, the member of the House proposing the motion is negative about the consideration concept node (i.e. closing of the prison with [very] limited guarding), and positive about the request concept node. The position of the government concerning the consideration and the request concept node cannot be discerned from the text of the motion itself, but needs to be reconstructed from the transcriptions of the debate preceding the voting stage [23]. In the example motion, the government is negative about the request part (i.e. it does not want to postpone the decision and keep the limited guarding prison places), because the motion was based on incomplete and incorrect information. From the transcripts of the statements made by the Secretary, it can further be inferred that the government is positive about the intended abolishment of limited guarding, i.e. there is a positive link of the government and the consideration concept variable.

Fourth, determining the value of the four links between the two concept variables (i.e. the consideration part and the request part) and the two utility nodes (i.e. the House and the Government) represents the core of the coding process (see [23], for a detailed description of the procedure). The underlying template for analyzing statements is the following [23]: The proposer's / government’s attitude towards the present/improved view is negative / positive. The overall result of these first four steps is a graph (or cognitive map), consisting of concept variables, utility nodes, causal relations between the concept variables, and signed relations between concept variables and utility nodes.

In a final step, social dilemma structures are identified. In order to do this, it is necessary to first define how the four types of social dilemmas translate into different cognitive maps. The rationale for this step is in detail outlined in [6]. We use two of the cognitive maps they proposed (Coordination and Prisoner’s Dilemma), and add two new structures (Battle of the Sexes and Pure Competition). Note that in theory a single motion may contain more than two concept variables, and therefore may actually be composed of several different social dilemmas. This may be problematic where the dilemmas differ. As argued before, in practice motions contain two concept variables. Consequently, each motion could be unambiguously linked to one specific social dilemma. Fig 2 presents the graphical presentation of all four types of cognitive maps. In the example motion, the proposer is negative about the consideration part and positive about the request part, whereas the government is positive about the consideration part and negative about the request part. Hence, the underlying structure of the example motion is a pure competition social dilemma.

### Measures

Our dataset consists of one dependent variable (acceptance of a motion), seven independent variables, and one control variable.

#### Acceptance

The dependent variable *Acceptance* was coded “1” if the majority of the members of the House of Representatives voted in favor of the motion. This information is publicly available on www.overheid.nl. The number of votes in favor of the motions might also have been used as dependent variable. We prefer a dichotomous outcome for two reasons. First, the decisive criterion for a motion is whether or not it is passed. The number of votes also depends on the number of parliamentarians participating in the voting, and this number fluctuates considerably because not all parliamentarians show up during all votes. In addition, a linear regression with number of votes as dependent variable (not reported), revealed virtually the same results as our logistic regression, with almost the same ratio in values found for the dependent variables.

#### Proposer

The variable *Proposer* was coded “1” if the main proposer of the motion was a member of a coalition party, and “0” if it was a member of the opposition.

#### Number of proposing parties

For each motion, the number of parties proposing it was counted.

#### Voting potential

For each motion, it was determined which parties supported it during the proposal stage. Subsequently, the number of members in the House of Representatives pertaining to these parties were counted. The variable *voting potential* consists of this count.

#### Coordination game

The motion was coded “1” if the underlying cognitive map represented a coordination game and “0” otherwise.

#### Battle-of-the-sexes game

The motion was coded “1” if the underlying cognitive map represented a battle of the sexes game, and “0” otherwise.

#### Prisoner’s dilemma game

The motion was coded “1” if the underlying cognitive map represented a prisoner’s dilemma game and “0” otherwise.

#### Pure competition game

The motion was coded “1” if the underlying cognitive map represented a pure competition game and “0” otherwise.

#### Cabinet

During the time covered by the available motions the Netherlands had two different governments. The first one in 2009–2010 was based on a coalition of Christian and social democratic parties. This is the Balkenende IV government. The second one, the Rutte government, was based on conservatives and Christians and had a supply agreement with an anti-Islamic nationalistic party. This party is considered as a coalition party. In order to control for the possibility the type of cabinet affects the acceptance of motions, we created the control variable *Cabinet*. It was coded “1” if the motion was proposed during the first period (the Balkenende cabinet), and “0” if it was proposed during the second period (the Rutte cabinet).

Two independent coders coded the material. Coders were ignorant about the hypotheses to be tested in this study. Each coder had to make five coding decisions per motion [23]: for both the Government and the House, the coder had to determine the position with regard to the two concept variables (i.e. is the effect on their utility negative or positive). The fifth decision consists in determining the argument that the government takes with regard to the request concept node. Both coders were trained by first independently coding thirty motions that had been withdrawn and which therefore are not part of our sample of 822 motions. The first author also coded these thirty motions. Inter rater reliability during this trial phase varied between π = .71 for coding the consideration part according to the government, and π = .87 for coding the request part according to the government. Subsequently, the reasons for disagreement were discussed and resolved. In a second step, both coders subsequently received small subsamples of the whole set of motions and coded them. Two coders coded half of the set; one half was coded by only one coder. Inter rater reliability for the real sample for the two coders ranged between π = .85 for the consideration part, and π = .96 for the request part according to the government.

The index π [24, 25] is appropriate when a large number of assignments among trained coders have to be compared. It does not assume a priori differences between the coders, and computes deviations from expected agreement by using the marginal distribution over all coders.

## Results

The descriptive results are presented for the 700 sampled motions that were actually put to vote. On average motions are proposed by just over 2 parties, and the number of votes these parties have is 37. This is not half of the number 76 needed to get the motion passed. The average number of votes in the voting however is 80. This shows that in general the proposer can have good hope that their motion also finds support among colleagues. Motions proposed by coalition parties are usually supported by 2 parties. For motions proposed by opposition parties the average number of parties supporting it is 2.7. Average voting potential for motions of the coalition is 30, compared to an average of 60 for opposition parties.

Of the 122 motions in the sample that were not voted for, the opposition proposed 90. Here, the distribution of social dilemmas consists of 8 coordination games, 34 battle of the sexes, 6 prisoner’s dilemma, and 74 pure competition. Looking at the motions that did not reach the voting stage 35 had the support of the government or the action requested was already taken or initiated, 32 were premature, 7 in conflict with the existing law and 9 in conflict with earlier agreements in the House.

With regard to the outcomes of the voting, four notable patterns can be discerned (see Table 1). Note, we use game theoretic *representations*, we do not perform a game theoretic *analysis*.

**Table 1:** Frequency of Types of motions submitted per main proposer and the outcome of the voting.

	Coalition party		Opposition party		
	Motion Accepted	Motion Rejected	Motion Accepted	Motion Rejected	Total
Coordination game	79	0	55	19	153
	(52.7%)	(0%)	(36.2%)	5.1%	21.9%
Battle of the sexes game	27	0	34	13	74
	(18%)	(0%)	(22.4%)	(3.5%)	(10.6%)
Prisoner dilemma game	18	2	23	70	113
	(12%)	(8.3%)	(15.1%)	(18.7%)	(16.1%)
Pure competition game	26	22	40	272	360
	(17.3%)	(91.7%)	(26.3%)	(72.7%)	(51.4%)
Total	150	24	152	374	700

Column percentages in brackets.

First, though opposition parties proposed three quarters of all motions, motions proposed by a coalition party had a far higher chance of getting accepted than motions proposed by the opposition: of the 174 motions proposed by the coalition, 86.2% (150) were accepted, compared to 29% (152 out of 526) proposed by the opposition. All motions proposed by the coalition that had the support of the government passed the vote. This is only true for 74% (89) of the motions proposed by the opposition. 25% of the motions proposed by the opposition that were rejected were proposed too early (the government was positive, but was waiting for specific data that were not yet available), and the action requested in one of the motions (3%) has already been taken (according to the government). 75% of these rejected motions took place under the Rutte government.

Second, about half of all motions that came to the vote (51%) are based on a pure competition dilemma, followed by coordination games (22%), prisoner’s dilemmas (16%) and battle of the sexes (11%). Coalition and opposition parties proposed a similar number of coordination motions, but differ considerably with regard to the number of prisoner’s dilemma, pure competition, and battle of the sexes motions, which are proposed far more often by the opposition.

Third, motions with a coordination or battle of the sexes structure were far more likely to pass than motions with a prisoner’s dilemma or a pure competition structure. All coordination or battle of the sexes motions that were proposed by a member of a coalition party have passed, whereas the percentage of acceptance for these two types of motions drops to 74% ((34 + 55) / 121) if they were proposed by a member of the opposition. Of all four types of motions, pure competition motions were indeed least likely to get accepted.

Logistic regression is applied, with the outcome of the vote as the dependent variable (reference category is rejection of the motion). Five models were estimated (see Table 2). In each model one or more independent variables are entered in line with the theory: first the control variable (the cabinet); next whether or not the main proposer is a member of a coalition party; third, the variables related to the voting power; and finally the game theoretic representations.

**Table 2:** Results of Logistic Regression of Voting Success During Motions on Type of Dilemma and Voting Power. Dependent variable: outcome of the voting on a motion (reference: not passed).

	Model 1	Model 2	Model 3	Model 4	Model 5
Cabinet	-.134	-.347*	-.182	.134	
Main proposer from coalition party		2.775*	2.100*	1.947*	2.009*
Number of proposing parties			-.009	-.067	
Voting potential			.032*	.035*	.031*
Battle of sexes game				.127	
Prisoner’s dilemma game				-2.186*	-2.106*
Pure competition game				-3.168*	-3.090*
R^2^	.001	.32	.42	.62	.62

* = significant at the .05 level, N = 700

Model 1 contains only the control variable *Cabinet*. It shows that the type of cabinet (Balkenende vs. Rutte) does not significantly affect the likelihood of acceptance or rejection of a motion.

Model 2 adds the party membership of the proposer, one of the three power variables. It shows a strong significant and positive effect for this variable, but also a small significant negative effect for the variable *Cabinet*.

Model 3 adds the two other power variables: the number of proposing parties has no significant effect, whereas voting potential has a small significant and positive effect on the likelihood of acceptance of a motion. In this model, the effect of *Cabinet* is not significant. The explained variance of model 3 is 42%, which means a sizeable increase compared to the 32% of variance explained by model 2.

Model 4 adds the social dilemma variables, with the coordination game being the reference category. It reproduces the effects of model 3. In addition we find a strong and significant negative effect for prisoner’s dilemma motions, and an even stronger effect for pure competition motions. Battle of the sexes motions do not have a significant association with the likelihood of a motion being accepted. Explained variance of model 4 is 62%, which again is a sizeable increase compared to model 3.

Model 5 represents the final model, in which the non-significant variables of model 4 were taken out. The effects remain similar to those in model 4. Overall, it shows that effect sizes for the two social dilemma variables are stronger than the effect sizes for the two power variables. The strongest predictor in the model is the pure competition dilemma, followed by the prisoner’s dilemma and the party of the main proposer. Voting potential affects the likelihood of passing, but the effect is small.

Based on model 5, the following conclusions can be drawn with regard to the two hypotheses. First, the data partially support the voting power hypothesis: motions proposed by the coalition are 7.45 times more likely to be accepted than votes proposed by the opposition, thereby supporting H1a. The number of parties supporting a motion proposal does not significantly affect the success or failure of a motion in the voting stage, which leads us to reject H1b. Finally, the positive and significant effect of voting potential is in line with H1c.

Second, our data provide strong support for the social dilemma hypothesis, which suggested and inverse relationship between the severity of the social dilemma and the likelihood of a motion to be accepted. More specifically, we found that compared to coordination games, battle of the sexes games do not significantly affect the likelihood of a motion to be accepted or rejected. As predicted, we found strong negative effects for prisoner’s dilemma and pure competition games: prisoner’s dilemma motions are 8.2 times less likely to be accepted than coordination motions. This figure almost triples for pure competition motions, which are 21.8 times less likely to be accepted than motions reflecting a coordination game. Hence, as predicted, we see a gradual decrease of the likelihood of acceptance along the continuum of severity of the social dilemma structures underlying the motion.

During the Rutte legislature, one of the parties had a supply agreement with the coalition. In our analysis, this party is coded as a coalition party in the variable “proposer”. However, for some issues, parties with supply agreements may take different policy positions and its voting behavior may deviate from the position of the coalition. In order to test whether grouping the supply party as a coalition member affected the outcome of our statistical tests, an additional analysis (not reported) was performed. Here, the supply party was coded as a separate (third) category of the variable “proposer”. Results of this statistical analysis turned out to be in line with the other models.

## Discussion and Conclusion

Motions are an important element of political control in parliamentary democracies, and a key instrument to correct or change specific government policies. Yet, why some motions get accepted and others don’t so far has attracted only limited attention in the scholarly literature. Our study aimed to tackle this gap. In line with much earlier research, we found that voting power matters: the chance that a motion passes the voting stage is much higher if a member from the coalition party proposes it, and if the voting potential is high. But our study also showed that the severity of the social dilemma behind the motion matters even more: requests that reflect an underlying conflict situation—i.e. a zero sum or prisoner’s dilemma problem—are far less likely to be accepted than motions reflecting a more cooperative situation, i.e. coordination or battle of the sexes problem. In fact, the explanatory power of the type of social dilemma outweighs the other variables in our model.

Our study and its findings enrich current scholarship in at least three important ways. First, up until now, with the exception of a handful of studies (e.g. [1, 3, 4) parliamentary motions only recently have attracted attention as a powerful subject for systematic quantitative analyses of collective decision-making. We build on this emerging tradition through the creation of a unique dataset of a stratified random sample of 822 motions as they were proposed in the Dutch Parliament from September 2009 to February 2011.

Second, we enrich theoretical modeling of political negotiations through complementing existing power based explanations by a social dilemma explanation. According to this perspective, the potential success or failure of a motion during the voting phase can be predicted by its underlying social dilemma structure, independently of the relative power of the involved players. We found strong evidence for this perspective, which indicates that it at least equals, if not outperforms conventional power based explanations. Jointly, both approaches explain more than 60% in the variance of the success or failure of the motions in our sample.

Third, we show that cognitive mapping techniques are a powerful analytical tool to extract social dilemma structures from highly codified political texts. Here, we draw on and extend a methodological innovation that was first introduced in [6] in an analysis of transitivity violations in the argumentation patterns in the so-called Federalist Papers. The method was subsequently applied to uncover how social dilemma structures change in the course of an organizational transition [7]. In both cases, coding was applied to unstructured text, like newspaper articles, pamphlets and flyers or interview transcripts. Our study shows that parliamentary motions represent an ideal setting for the investigation of social dilemmas in political negotiations for at least three reasons: due to the requirement to follow a predefined template, they represent highly structured and standardized units of analysis; the set of relevant stakeholders is clearly defined and their political positions allows to infer how specific concept variables may affect their utility; there is a clearly defined (binary) outcome variable (acceptance vs. rejection). The highly codified nature increases comparability between motions, and facilitates systematic coding.

Some open questions remain. They can be related both to the study of motions as to the representation of the underlying social dilemmas. First, 77% of the motions proposed by the opposition have a Prisoner’s dilemma or Pure Competition structure (compared to 39% of the motions proposed by the coalition), yet only 16% of these motions got accepted (compared to 65% success rate for these two types of motions for the coalition). Why would an opposition party propose such motions, assuming that proposers are rational and therefore have an intuition that such types of requests have a low chance to get accepted? Such proposals may still make sense if acceptance of the motion is not the primary objective of the proposer. Indeed, motions may be proposed for a variety of strategic reasons, like a party’s intention to signal to its voters and members that a specific issue is high on her agenda, or to test out the reaction of the adversary. Our social dilemma approach may inform future studies by taking into account a party’s motives for proposing a motion [9], and in particular the salience it attaches to the underlying issues. Coding of party manifestos may offer a fruitful avenue in this respect [26].

Second, the current study relied exclusively on the texts of the motions in order to determine the underlying social dilemma. An untested assumption in this approach is that the involved players—i.e. members of Parliament—indeed perceive these underlying structures and base their voting behavior on it. Future research might benefit from a more in-depth investigation of how variations in social dilemma structures in parliamentary motions are actually perceived and interpreted by political players.

Third, the combination of cognitive mapping and utility reasoning proved to be a powerful tool to extract a limited set of social dilemmas from texts. However, as long as the investigator needs to rely on assumptions about payoffs, this technique reaches its limits when it comes to disentangle more fine-grained differences between social dilemmas.

Fourth, our study is limited to parliamentary motions in the Netherlands, during a very specific period in time. As is the case for the voting power perspective, the underlying assumption is that the theoretical mechanism behind the social dilemma perspective holds across institutional contexts and historical periods. But in order to substantiate such a claim, comparative studies in different political systems and during a variety of historical periods would be required.

Finally, the main principle behind the social dilemma approach—i.e. that there is an inverse relationship between the severity of the social dilemma and the likelihood of a cooperative outcome, and that this relation holds independently of power structures—should hold also for other kinds of negotiations, both within and outside the political realm. As such, the core idea is neither tied to the specific domain of parliamentary motions, nor to the specific cognitive mapping techniques used in our study. As a general principle, this social dilemma perspective may fruitfully complement the more widespread actor and power centered approaches that currently inform most of our understanding of cooperation and conflict in politics and society.

## Appendix: Eliciting Social Dilemmas from Texts on Parliamentary Motions

All motions criticize the status quo, and suggest that a much better situation will result if the actions asked in the request are indeed carried out. This implies a negative relation between the status quo and the desired new situation. Consequently, the utility of the motion for the proposer is always negative with respect to the status quo, and positive with respect to the desired situation (as part of the request). The utility for the government can differ in both situations: it can agree or disagree with the description of the status quo, and it can agree or disagree with the request.

As a result, four different social dilemma structures are possible. We refer to them as coordination, battle of the sexes, prisoner’s dilemma and pure competition. In this Appendix, we present an example motion for each of these four structures. In addition, we summarize the advice given by the government in reaction to the motion. This information was from the transcription of the debates related to each motion. For a more detailed methodological description of the coding procedures related to extracting social dilemma structures from parliamentary motions, see [23].

### Exhibit 1: Coordination Motion

Example: Motion 32500-x, nr. 31; dated November 29, 2010:

The House, after hearing the deliberation,

- considering that virtually all European allies in EU and NATO are currently forced by circumstances to effectuate substantial structural cuts in their armed forces;
- also considering that the already limited effectiveness of the joint European defense efforts thus will decrease further;
- believing that this trend can be reversed by increasing efforts to intensify the mutual defense cooperation, such as pool formation for large transport capacities in water and in the air, further job specialization and the realization of an internal market for defense products within the EU;
- believing that sovereignty and control over the deployment of the Dutch armed forces remains fully intact;

requests the government in the shortest possible time to take initiatives to achieve such intensified structural cooperation with the European partners and to add concrete options for this purpose in the policy letter that has been promised to the House in next spring, and proceeds to the order of the day.

The advice by the Secretary is clear: “We will do that.” But he adds that he cannot promise whether the new commitments will already be added in the letter of policy to come. Both the House and the government agree that the present situation is not wanted and that what is suggested is good or at least better than the present situation.

### Exhibit 2: Battle of the Sexes Motion

Example: Motion 32500-xv, nr. 41; dated December 9, 2010.

The House, after hearing the deliberation,

- considering that the pension reserve has a deficit of 200 billion Euro;
- considering that the government wants to balance funding and certainty of pension rights by adapting the Pension Act and the Financial Assessment Framework for Pension (FTK);
- requests the Government to facilitate, through these amendments, a generation neutral solution of the reserve deficits in order to ensure that one generation does not cause structural payment increases to the pensions of another generation;

and proceeds to the order of the day.

The Secretary agrees with a generation neutral solution. He claims that the current recovery scheme already takes the interests of young people into account, and that the present scheme does not just benefit current pensioners. In case the proposer is willing to accept that this is true, the Secretary has no problem with this motion should it be accepted. According to the House the present situation has to improve, but according to the government this improvement has already started and will continue. The House learned that a new improvement (policy) had already started, but it can also be that the Secretary was willing to rephrase the original view so that at the end both parties agree. It remains unclear which view is correct, the fact whether the motions comes to the vote might give an indication. Most important is that both parties agree.

### Exhibit 3: Prisoner’s Dilemma Motion

Example: Motion 21501–07, nr. 783; February 17, 2011.

The House, heard the deliberation,

- noting that the crisis in the euro in part started because no adequate preventive intervention on the budgetary and economic imbalances occurred;
- noting that the European Union has not sufficient competences to enforce credible reforms;
- noting that the budget law of the House covers the emergency fund;
- requests the Government to agree only with an extension of the emergency fund when alternate arrangements will be linked that tackle economic imbalances and increase competitiveness of the European Union,

and proceeds to the order of the day.

The Secretary admits there are good points, but nevertheless he discourages the motion. It contains some elements that are not clear, for example that there are not sufficient competences to be able to demand credible reforms. The Secretary suggests that at the end he will only agree with a proposal after it has been discussed and permission is given in case the parliament has to approve. For this reason he will make a parliamentary scrutiny reservation on such matters that concern the budget law of this House. The government does not contradict the evaluation of the present situation, but also is not willing to accept to suggestion in the motion.

### Exhibit 4: Pure Competition Motion

Example: Motion 32563, nr. 2; December 8, 2010.

The House, after hearing the deliberation,

- ascertaining that the opinion of the committee *International Committee Management Oostvaardersplassen* [name of an area] (IMCO) reads that 500 ha of Hollandse Hout [name of an adjacent area] in winter is to be open to the large grazers and red deer from the Oostvaardersplassen for shelter;
- considering that the management by the State Forestry at the Oostvaardersplassen in recent years has led to unnecessary animal suffering and degradation of different bird populations and of water quality;
- ascertaining that keeping too many large herbivores in the Oostvaardersplassen resulted in permanent damage to the tree populations and decrease in the variation of vegetation structure;
- considering that, the opening up of the Dutch timber for large herbivores entails restrictions for recreational persons, resulting in permanent damage to the tree population, resulting in higher costs to maintain the water quality and entails risks to road safety;
- requests the Government not to permit large herbivores and deer in Hollandse Hout, but instead to take care of shelter measures in the existing Oostvaardersplassen area, and proceeds to the order of the day.

In the advice the minister explained that the committee had very good arguments and that these were followed. He agreed with the local authorities of the most nearby town that they will investigate whether there is sufficient support for the present measure: locally, municipally and among the citizens. The Secretary wants to wait until the results are known and does not want to disturb this process. For this reason he does not support the motion. The utility of the government is that what is going on is good. This does not hold for what is suggested in the motion, so there are opposed opinions, there is a conflict.
